# Antiracism in Action: Development and Outcomes of a Mentorship Program for Premedical Students Who Are Underrepresented or Historically Excluded in Medicine

**DOI:** 10.5888/pcd20.220362

**Published:** 2023-06-15

**Authors:** Fatuma-Ayaan B. Rinderknecht, Aminta Kouyate, Semhar Teklu, Monica Hahn

**Affiliations:** 1 University of California, San Francisco, School of Medicine; 2 University of California, Berkeley

## Abstract

**Introduction:**

Black, Latinx, and Native American and Alaska Native people are underrepresented in medicine. The increasingly competitive medical school application process poses challenges for students who are underrepresented in medicine or historically excluded from medicine (UIM/HEM). The University of California, San Francisco–University of California, Berkeley (UCSF–UCB) White Coats for Black Lives Mentorship Program provides a novel and antiracist approach to mentorship for these premedical students.

**Methods:**

The program recruited UIM/HEM premedical and medical students through a survey advertised by email, on the program’s website, social media, and by word of mouth. The program paired students primarily with race-concordant mentors, all of whom were UCSF medical students. From October 2020 to June 2021, program mentees engaged in skills-building seminars based on an antiracism framework and received support for preparing medical school applications. The program administered preprogram and postprogram surveys to mentees, which were analyzed via quantitative and qualitative methods.

**Results:**

Sixty-five premedical mentees and 56 medical student mentors participated in the program. The preprogram survey received 60 responses (92.3% response rate), and the postprogram survey received 48 responses (73.8% response rate). In the preprogram survey, 85.0% of mentees indicated that MCAT scores served as a barrier “a great deal” or “a lot,” 80.0% indicated lack of faculty mentorship, and 76.7% indicated financial considerations. Factors that improved most from preprogram to postprogram were personal statement writing (33.8 percentage-point improvement, *P* < .001), peer mentorship (24.2 percentage-point improvement, *P* = .01), and knowledge of medical school application timeline (23.3% percentage-point improvement, *P* = .01).

**Conclusion:**

The mentorship program improved student confidence in various factors influencing the preparation of medical school applications and offered access to skills-building resources that mitigated existing structural barriers.

**Summary**

**What is already known on this topic?**

While the US becomes increasingly diverse, Black, Latinx, Native American and Alaska Native, and other racial and ethnic minority groups remain underrepresented among US physicians. This disparity is associated with poor health outcomes in racial and ethnic minority populations.

**What is added by this report?**

Many previous interventions and mentorship programs aimed to diversify the health care workforce. We describe a novel and successful mentorship program run by racial and ethnic minority medical students and centered on antiracism in medicine.

**What are the implications for public health practice?**

Similar programs are likely to improve numbers of racial and ethnic minority physicians and reduce racial health disparities.

## Introduction

Over the last century, despite the increasingly diverse US population, racial and ethnic diversity in the medical profession is stagnant. For example, the number of Black men who applied to medical school was lower in 2014 than in 1978 (1). As a consequence of historical and contemporary inequitable policies that systematically exclude racial and ethnic minority applicants, racial discordance persists. Black, Latinx, and Native American and Alaska Native people make up 5%, 5.8%, and 0.3% of US physicians, compared with 13.4%, 18.5%, and 1.5% of the US population, respectively (2,3).

These disparities result, in part, from historically racist policies, such as those resulting from the Flexner report (4). Published in 1910 to set medical education standards and practices, this report had a devastating effect on the racial makeup of medical schools and ultimately resulted in the closure of 13 historically Black colleges and universities that had been established between 1865 and 1904. An economic analysis of the effects of these closures determined that if this report had not been published, an additional 35,315 Black medical professionals would have been in the health care workforce in 2019 (4).

Racial discordance in the medical field likely perpetuates racially homogenous medical research, a lack of access to health care, and health disparities that disproportionately affect racial and ethnic minority groups (5). Black people in the US have the lowest life expectancy, and compared with their White counterparts, fare worse in maternal and childbirth outcomes and have higher rates of cancer, stroke, and hypertension (6). Increasing the number of racial and ethnic minority physicians would likely have a positive effect on reducing health disparities. Black and Latinx patients have a higher level of satisfaction with a racially concordant physician than with a physician from a different race (7), and physicians who are members of medically underserved racial or ethnic minority groups (Black, Latinx, or Native American/Alaska Native) are more likely than physicians who are not from medically underserved minority groups to provide health care to medically underserved populations (8).

Although mentorship programs exist for medical students who are underrepresented in medicine (UIM), or are “historically excluded in medicine” (HEM), few are run by medical students or physicians who identify as UIM/HEM. Moreover, to our knowledge, no current mentorship programs offer truly reparative solutions that mitigate structural barriers to navigating the medical school application process for racial and ethnic minority students.

The University of California, San Francisco–University of California, Berkeley, White Coats for Black Lives Mentorship Program (UCSF-UCB WC4BL) provides a novel and antiracist approach to mentorship and the development of pathway programs for UIM/HEM premedical students. The program uses antiracism as a framework for health professionals and trainees that incorporates critical perspectives to prepare individuals to directly address the root causes of race-based disparities in medicine and health care (9,10). This antiracist framework was operationalized through a series of seminars and workshops that incorporated the topics of restorative justice, multilevel systems of oppression, and imposter syndrome. Imposter syndrome is defined as “feelings of inferiority regardless of one’s accomplishments and experiences. Imposter syndrome is often viewed as an experience that racially minoritized populations in higher education [encounter]” (11). Incorporating antiracism into the mentorship program included matching mentees with racially concordant mentors, comprehensive discussions on how systemic racism affects health care, scholarships for UIM/HEM premedical students, and tools on how to maintain well-being as racial and ethnic minority trainees in the medical field. The objective of our research was to describe the outcomes and lessons learned from this pilot program as a blueprint for antiracist UIM/HEM pathway programs.

## Methods

### Selection of mentees

The UCSF-UCB WC4BL medical-student leadership board selected UIM/HEM premedical and prehealth students or graduates from October 2020 to August 2021 for a year-long mentorship program organized by the UCSF-UCB chapter of the national WC4BL organization (www.whitecoats4blacklives.org). Potential mentees submitted applications, which included short personal statements and resumes, in August 2020. Volunteer mentors were UCSF medical students recruited in August 2020 from UIM/HEM student groups such as the Latino Medical Student Association, the Student National Medical Association, and other groups. The UCSF-UCB WC4BL leadership board, consisting of UIM/HEM medical students, reviewed all applications. 

Eligibility criteria for mentee participation were 1) being a person from a UIM/HEM group as defined by the UCSF Office of Diversity and Outreach, with the option to self-identify, and 2) being on the premedical or prehealth track with at least first-year standing in an undergraduate institution (12). All applicants were accepted.

### The mentorship program

Mentees were required to meet virtually with their mentors at least once per month from October 2020 through June 2021 and attend seminars to broaden their perspective on the field of medicine and learn key skills and information to be successful premedical students. Additionally, mentees were tasked with writing an op-ed that addressed an issue at the intersection of racism, health equity, and medicine, and in alignment with the mission of the program, they were asked to describe a plan of action for actualizing racial equity. This op-ed was an opportunity for students to apply the knowledge gained throughout the seminar series on antiracism in medicine and develop strategies and novel ideas to address structural issues affecting historically marginalized populations. At the start of the program, a virtual orientation was held, and instruction on op-ed writing was facilitated through a workshop. The UCSF-UCB WC4BL leadership board developed and coordinated virtual seminars on the medical school application process and other topics that were determined to be important for student success (Table 1). Mentees were also connected to clinical research and volunteer opportunities with the vaccine distribution program at UCB University Health Services to provide them with experiences vital to becoming successful medical student applicants.

**Table 1:** Monthly Seminar Series for Participants in White Coats for Black Lives (WC4BL) Mentorship Program, University of California, San Francisco–University of California, Berkeley, 2020–2021

Seminar topic	Learning objectives	Presenter
**Fall 2020 programming**		
Medical school application overview	• Review the multiple components of medical school applications such as general prerequisites, general application timeline, personal statements, letters of recommendations, MCAT • Identify scholarships and resources to afford costs of medical school applications	UCSF medical students
UCSF–UCB WC4BL UC PRIME Mentorship Conference	• Provide a networking opportunity for UC PRIME medical students and premedical undergraduate students • Foster dialogue on racism as a public health concern and discuss how to end racial discrimination in medical care • Prepare future physicians to be advocates for racial justice • Provide a framework for a successful mentee–mentor relationship	UCSF medical students
MCAT 101	• Review study strategies and organizational practices for MCAT readiness • Discuss paid and free MCAT study resources	UCSF medical students
Personal statements workshop	• Elucidate typical personal statement questions • Learn how to effectively write about personal experiences and difficult events • Provide a framework on asking for and implementing feedback	UCSF medical students
Open office hours	• Provide a space to ask questions about medical school applications, personal statements, the MCAT, and other topics • Receive feedback on writing pieces and personal statements	UCSF medical students
**Spring 2021 programming**		
AMCAS activities section overview	• Explore the significance of the “most meaningful activity” on AMCAS and strategize how to maximize writing about one's extracurricular activities • Learn how to engage in extracurricular activities during the pandemic	UCSF medical students
Nontraditional pathways and belonging	• Understand the differences between formal and informal postbaccalaureate programs • Identify and combat imposter syndrome	UCSF medical students
AMCAS overview	• Review the components of AMCAS • Examine resources to organize personal statements, letters of recommendation, and more	UCSF medical students
UC Berkeley public health literature and research seminar	• Learn how to approach public health literature research from a UC Berkeley Public Health librarian • Understand the tools to conduct literature research and analysis	UC Berkeley Public Health librarian
PRIME-US and WC4BL premedical conference	• Participate in a half-day conference catered to all groups of premedical students • Understand how you want to share a difficult topic into your application • Reframe the narrative of who we are as underrepresented minority students • Compare the pros and cons of a gap year between undergraduate and medical school • Break down the different components of the medical school application process	Keynote speakers: Leticia Rolón, MD, and Ronald L. Copeland, MD, FACS Physicians and UCSF medical students

Abbreviations: AMCAS, American Medical College Application Service; MCAT, Medical College Admission Test; PRIME-US, Program in Medical Education for the Urban Underserved at UCSF; UCB, University of California, Berkeley; UC PRIME, University of California Programs in Medical Education; UCSF, University of California, San Francisco; URM, underrepresented minority.

The UCSF-UCB WC4BL program was made possible through The Big C (Big Community) Fee Referendum at UCB, which provides funding for student-initiated projects (13). Because this program was conducted for educational program quality improvement purposes, and not for research purposes, it did not require institutional review board review or approval.

### Surveys

Mentees were sent 2 electronic surveys via email: a preprogram survey, administered on October 24, 2020, and a postprogram survey, administered on June 5, 2021, after completion of the program. The preprogram survey collected information on socioeconomic and demographic characteristics, including social and economic identity, gender identity, and racial and ethnic identity, and was used to pair mentees and mentors with similar interests and racial concordance. Both surveys asked about the extent to which certain factors posed barriers to their application to medical school and the extent of their confidence in certain aspects of their applications. Answer options were provided on a 5-point Likert scale ranging from “a great deal,” “a lot,” “a moderate amount,” and “a little” to “none at all.” Answer choices of “a great deal” and “a lot” were grouped together. Part of the postprogram survey used an adapted version of the Johns Hopkins Mentorship Effectiveness Scale (14). One question asked about stereotype threat, defined as the risk of confirming a negative stereotype about an individual’s background such as their race, gender, or ethnicity (15). Lastly, the presurvey asked 4 open-ended questions: 1) What do you think is the biggest obstacle to developing an ideal mentor–mentee relationship? 2) How can your mentor best serve you? 3) What do you think most mentorship programs lack? and 4) Questions? Comments? Concerns? 

### Quantitative analysis

Data were collected, analyzed, and stored in Qualtrics (Qualtrics Software Company). For each question on confidence and barriers, we calculated the percentage of respondents who answered “a great deal” or “a lot.” We linked data from the preprogram survey and postprogram survey and calculated percentage-point changes in confidence and barriers. We used the test of equal or given proportions in R studio (RStudio Team) to determine significant differences from preprogam to postprogram; *P* ≤.05 was considered significant. We also used this test to determine differences in demographic characteristics between our cohort and national data.

### Qualitative analysis

Two independent coders used a latent, inductive approach to thematic content analysis and coded responses to the open-ended questions. Discrepancies in coding were resolved through team meetings. Several themes were identified by the coders, and the total number of responses that fit into these themes was recorded.

### Ongoing program needs assessment

Given that this was a novel program, leadership continuously engaged mentees through conversations on mentorship needs that were not being met throughout the year. As program facilitators, it was necessary to be flexible and responsive to our cohorts’ needs and barriers. We noted these factors informally.

## Results

The mentorship program consisted of 65 premedical mentees and 56 medical student mentors. The preprogram survey received 60 responses (92% response rate), and the postprogram survey received 48 responses (74% response rate).

### Mentee characteristics

Of the 60 mentees who responded to the preprogram survey, 24 (40.0%) identified as Black and 23 (38.3%) as Latinx (Table2), which was almost 4 times the percentage of Black and Latinx medical student applicants in the US (8.7% and 9.5%, respectively) (16). Fifty-five (92%) students in our cohort were UIM/HEM students. 

**Table 2:** Characteristics of Participants in White Coats for Black Lives Mentorship Program, University of California, San Francisco–University of California, Berkeley, 2020–2021^a^

Characteristics	No. (%) (n = 60)
**Social and economic identity**	
Low socioeconomic background	26 (43.3)
First-generation college student	26 (43.3)
LGBTQIA+	10 (16.7)
**Gender identity (pronouns)**	
She/her series	55 (91.7)
He/him series	5 (8.3)
**Racial and ethnic identity^b^**	
Black	24 (40.0)
Latinx	23 (38.3)
Filipinx/a/o	6 (10.0)
Non-Hmong, Non-Filipinx/a/o, or non-Vietnamese Asian	4 (6.7)
Pacific Islander	2 (3.3)
Hmong, Vietnamese, Cambodian, Laotian, Thai, Burmese	2 (3.3)
Afro-Latinx	1 (1.7)
Southeast Asian (Nepalese)	1 (1.7)
Native American or Alaska Native	1 (1.7)
**Year in school**	
Freshman (college)	1 (1.7)
Sophomore (college)	7 (11.7)
Junior (college)	14 (23.3)
Senior (college)	7 (11.7)
Postbaccalaureate/masters	13 (21.7)
Nontraditional student	14 (23.3)
Other	4 (6.7)

Abbreviation: LGBTQIA+, lesbian, gay, bisexual, transgender, queer, intersex, asexual.

a Data collected from the preprogram survey; of the 65 students who participated in the program, 60 completed the preprogram survey.

b Participants could choose >1 race or ethnicity.

Our cohort had a significantly greater proportion of students from a low socioeconomic background when compared with all medical students in the US (17) (43.3% vs 6.0%; *P* < .001). Similarly, our cohort had a significantly greater proportion of students who were first-generation college students when compared with US medical school matriculants in 2018–2019 (18) (43.3% vs 10.8%; *P* < .001). Lastly, our cohort had a higher proportion of gay, lesbian, bisexual, transgender, queer, intersex, and asexual (LGBTQIA+) students when compared with the percentage of LGBTQIA+ medical students in the US (19) (16.7% vs 9.3%; *P* = .17).

### Barriers for UIM/HEM medical school applicants

Mentees reported many barriers to the medical school application process (Table 3). The factors that posed the greatest barriers to mentees in the preprogram survey were Medical College Admission Test (MCAT) scores, lack of faculty mentorship, and financial considerations: 85.0%, 80.0%, and 76.7% of students, respectively, indicated that these factors served as barriers a great deal or a lot.

**Table 3:** Preprogram and Postprogram Barriers and Confidence in Applying to Medical School and Career in Medicine Among Participants (N = 65) in White Coats for Black Lives Mentorship Program, University of California, San Francisco–University of California, Berkeley, 2020–2021

Survey question	Preprogram, %^a ^(n = 60)	Postprogram, %^a^ (n = 48)	Percentage-point change	*P* value^b^
**To what extent does the following serve as a barrier for medical school application?**				
My MCAT (current or anticipated) test scores	85.0	75.0	−10.0	.14
Lack of faculty mentorship	80.0	58.3	−21.7	.01
Financial considerations	76.7	72.9	−3.8	.63
My personal statement	73.3	39.6	−33.8	<.001
Awareness of medical school professors that look like me	68.3	45.8	−22.5	.02
Lack of peer mentorship	61.7	37.5	−24.2	.01
Racism (in any way you perceive this)	61.7	62.5	+0.8	>.99
Knowing the medical school application timeline	48.3	25.0	−23.3	.01
**Indicate the extent of your confidence in the following factors**				
I am confident that I can reach out to mentors throughout my journey.	55.0	72.9	+17.9	.01
I am confident in my knowledge of health equity.	53.3	77.1	+23.8	<.001
I am confident that I can identify personal feelings of stereotype threat.	48.3	72.9	+24.6	<.001
I am confident that I will apply the self-care techniques that I know, when I feel I need them.	41.7	64.6	+22.9	.002
I am confident that I will gain acceptance into medical school.	25.0	45.8	+20.8	.003
I am confident that I can identify all my personal strengths.	18.3	45.8	+27.5	<.001

Abbreviation: MCAT, Medical College Admission Test.

a Answer options were provided on a 5-point Likert scale ranging from “a great deal,” “a lot,” “a moderate amount,” and “a little” to “none at all.” Answer choices of “a great deal” and “a lot” were grouped together and calculated as percentages.

b Test of equal or given proportions in R studio (RStudio Team) was used to determine significant differences from preprogam to postprogram; *P* ≤.05 was considered significant.

The factors that improved most from preprogram to postprogram were as follows: personal statement development (33.8 percentage-point improvement, *P* < .001), peer mentorship (24.2 percentage-point improvement, *P* = .01), knowing the medical school application timeline (23.3 percentage-point improvement, *P* = .01), awareness of medical school professors who “look like me” (22.5 percentage-point improvement, *P* = .02), and faculty mentorship (21.7 percentage-point improvement, *P* = .01).

Many factors that had more than a 20.0 percentage-point improvement from preprogram to postprogram, such as personal statement development and knowledge of medical school application timeline, were reflected in the mentorship programming in seminars 1 (medical school application overview), 4 (personal statement workshop), 5 (open office hours), and 7 (nontraditional pathways and belonging).

### Factors affecting mentee confidence

The factors that mentees were most confident about in the postprogram survey were knowledge of health equity (77.1%), being able to reach out to mentors in their medical school journey (72.9%), and identifying personal feelings of stereotype threat (72.9%). The factors that mentees gained the most confidence in from preprogram to postprogram were confidence in finding mentors (28.3 percentage-point improvement; *P* < .001) and confidence in their ability to identify their personal strengths (27.5 percentage-point improvement; *P* < .001). The factor that mentees had the least amount of confidence in in the postprogram survey was knowing what to do when they felt they had imposter syndrome (33.3%).

### Key themes identified in qualitative analysis

We identified 5 key themes from the free-response text: 1) guidance through the medical school application process, 2) emotional support from mentors, 3) ability to be vulnerable with mentors, 4) tailored mentorship for UIM/HEM students, and 5) identity and race concordance (Table 4). For example, for the first theme, one mentee stated that they gained “insight into the application process . . . interview tips . . . and feedback on personal statement.” For emotional support from mentors, one mentee stated they were reminded “how to stay grounded and motivated with so much injustice and constant reminders [UIM/HEM premed students] are “not good enough” or “do not belong.” For the ability to be vulnerable with mentors, one mentee remarked they had difficulty with deciphering “when it is necessary to reach out to your mentor [while ensuring] you are not overbearing.” For identity and race concordance, for example, one mentee stated that they highly valued “[s]hared background/life experience that can help with relatability and feeling understood” as a key strength in the relationship with their mentor.

**Table 4:** Mentee Quotes Representative of Key Themes Identified by Cohort Analysis, White Coats for Black Lives Mentorship Program, University of California, San Francisco–University of California, Berkeley, 2020–2021^a^

Themes	Representative quotes	No. (%) (n = 60)^b^
Guidance through medical school application process	1) [I]insight into the application process 2) interview tips: especially tips on how to navigate microaggressions on the interview trail 3) feedback on personal statement: how much is too much to share related to traumas that have shaped my drive to pursue medicine 4) strengthen my “why medicine” pitch.	29 (48.3)
Emotional support	[H]ow to stay grounded and motivated with so much injustice and constant reminders we are “not good enough” or do not belong. . . [H]ow to feel valued and not “othered” when our values do not align with institutions.	23 (38.3)
	It has been difficult, to say the least, navigating this journey with minimal guidance. I applied to this program because this year I realized I cannot continue on this path alone. I had many breakdowns that I believe could have been prevented if I had mentors to turn to who can provide guidance and resources as I continue on my path to medical school. I can say with confidence that participating in this program will contribute to the fabric of my excellence, putting me on the right path to fulfilling my dreams of becoming a physician. This program will not only positively impact me, but also my community.	
Mentee anxiety about being vulnerable with mentor	From my personal experience, I believe often times no matter how welcoming or reassuring a mentor is, a mentee can feel [that] their curiosity comes across as overbearing, and they avoid asking any many questions they would like. It can be difficult to decipher at times when it is necessary to reach out to your mentor, but also ensuring you are not overbearing.	17 (28.3)
Tailored mentorship for UIM/HEM students	A better understanding of the premed and medical school experience as an underrepresented student	7 (11.7)
Identity mismatch	Shared background or life experience that can help with relatability and feeling understood.	5 (8.3)
	Mentors not looking like their mentee or not having anything in common to connect with.	

Abbreviation: UIM/HEM, underrepresented in medicine or historically excluded from medicine.

a A preprogram survey asked 4 open-ended questions: 1) What do you think is the biggest obstacle to developing an ideal mentor–mentee relationship? 2) How can your mentor best serve you? 3) What do you think most mentorship programs lack? and 4) Questions? Comments? Concerns?

b Number of students who provided an open-ended comment that fit with each theme. All 60 participants responded to the open-ended questions.

### Needs assessment

In response to informal conversations during the program year, WC4BL leadership developed new partnerships and seminars. A seminar series was launched on strengthening study strategies for the MCAT and a partnership was formed with the Princeton Review to provide mentees discounted MCAT preparation courses and an advisor at the Princeton Review who assisted mentees with planning their study strategy free of charge. Additionally, mentees were able to apply for scholarships from UCSF–UCB, and 5 mentees per semester were eligible to receive an additional stipend for contributions to program development. Because 43% of the mentees indicated being from a low-income background, this partnership and these scholarships were essential to alleviating a key structural barrier, financial disadvantage and resultant stress, while supporting students in their academic pursuits. Lastly, to address perceived barriers to faculty mentorship, we held 2 conferences during the year in which UIM/HEM faculty were invited to speak and connect with mentees, providing additional active support to students to mitigate structural barriers.

## Discussion

It is clear from the literature (1–3,8) and analysis of the UCSF-UCB WC4BL mentee experience that specialized mentorship programs that center the needs of UIM/HEM students are successful and necessary to recruit future diverse health care professionals.

Several factors in the approach of the UCSF-UCB WC4BL mentorship program were unique and tailored to the mentorship of UIM/HEM students. These factors included having a leadership team composed of UIM/HEM medical students who had intimate knowledge of the UIM/HEM premedical lived experience. This factor was essential to the program’s success in targeting the barriers UIM/HEM students face in their path to medical school. Additionally, the program used an antiracist framework, ensuring first that students were aware of key antiracism concepts such as power, privilege, and levels of oppression and intersectionality. The program also offered examples of systemic racism in the health care field and real-world first-hand narratives of UIM/HEM leaders in medicine, and equipped UIM/HEM students with the skills and self-care techniques necessary for successfully navigating a career in medicine. Furthermore, the program acknowledged and attempted to rectify structural barriers, such as lack of access to resources for MCAT preparation and access to racially concordant faculty mentors, through an equity-centered reparative approach.

This dynamic mentorship program was tailored to the needs of our mentees; we prepared preprogram surveys to understand what mentees identified as barriers in their medical school journey and created seminars based on this information. Additionally, we generated regular feedback throughout the year in discussions with mentors and mentees, to make changes to the program in real time.

Access to resources such as mentorship, knowledge of medical school application, and personal statement development were factors that improved the most from preprogram to postprogram. These findings suggest that our seminars, conferences, and one-on-one mentoring were effective in reducing mentees’ barriers to applying to medical school.

The 5 themes identified through our qualitative analysis showed that racial concordance and shared background experience between mentees and mentors were key to the success of the program as determined by the mentees. In addition to recruiting UIM/HEM mentors, we held 2 conferences that featured UIM/HEM physicians and offered opportunities for career and social networking. This programming was deemed successful by mentees, who reported they were significantly more likely after the program than before the program to feel confident there were physicians who looked like them. These findings highlight the importance of recruiting and using mentors and role models with some shared identities and understanding of the unique experiences of racial and ethnic minority students who are pursuing careers in medicine. 

The seminar series placed special attention on supportive messaging to prevent the possibility of imposter syndrome, provide guidance in the form of narratives from UIM/HEM medical students who had faced challenges in their own pathway to medical school, and offer novel strategies to find financial and material resources. However, we did not have seminars focused solely on financial barriers in medicine or how to diminish stereotype threat and imposter syndrome. The lack of this type of seminar may explain why financial issues and confidence in overcoming stereotype threat were 2 factors that did not improve as much as other factors from preprogram to postprogram. Future programming should create partnerships to provide additional resources in these areas.

From our partnership with the Princeton Review, we determined that it was essential to bring in outside expertise to provide tailored and detailed instructions for UIM/HEM students to be successful. In the future, we hope to partner with certified financial planners, mental health counselors that work with UIM/HEM in the health care field, and campus wellness resource groups. This strategy can contribute to the feasibility and sustainability of pathway development and mentorship programs by reducing strain on program leadership and stretching limited funding.

The mentorship program was made possible through UCB funding. Without continued funding, the program would not be sustainable. Our program relied on volunteers (ie, UIM/HEM medical students) to serve as mentors; future efforts must include financial support for these students, who are working to alleviate racial disparities and navigate their own future in medicine.

Lastly, simply increasing the numbers of UIM/HEM medical students is not enough to rectify the harmful effects of structural racism in the medical field. It is imperative that medical schools also take an antiracist approach in clinical and medical education. However, addressing this larger topic was outside the scope of our student-led initiative.

### Limitations

Our study has several limitations. First, our cohort had limited gender diversity; 91.7% identified with the she/her series. Moving forward, we intend to increase gender diversity, especially considering the decreasing numbers of Black men in medicine (1). Second, we had only 1 Native American/Alaska Native mentee, and we aim to increase this number through partnerships with Tribal colleges and universities. Third, we could not calculate exact numbers of racial concordance between mentors and mentees, because we did not record the number of racially concordant mentee–mentor pairs. Fourth, the postprogram survey response rate was 74%, possibly as a result of self-selection bias: those who completed the postprogram survey may have benefited more from the program than those who were lost to follow-up. Fifth, the sample size of 65 premedical students was small, and as such, we cannot claim that our results are generalizable to other populations. Finally, we do not yet have data on the number of program participants who have been accepted to medical school. Future research will follow this cohort and assess the success rate of our program.

### Conclusion

To achieve the goal of racial equity in medicine, programs like the UCSF-UCB WC4BL pathway development program are essential. These programs must have leadership teams composed of UIM/HEM medical students and professionals and implement programming informed by antiracist practices for UIM/HEM students to be fully supported in their medical school journeys.

Of course, pathway development and mentorship programs alone cannot solve the devastating consequences of centuries of exclusionary policies. As this unique program has done, medical institutions must approach this problem with a reparative justice lens (20), pairing acknowledgment of these past harms with substantive efforts to repair and redress these harms with resources and support for UIM/HEM students. By actively centering antiracism and providing material support in the form of financial aid and preparation for exams, more programs can actualize a reparative justice approach to enhance future workforce diversification and eliminate racism in health care and beyond.
